# Dysregulated lipid metabolism and intervertebral disc degeneration: the important role of ox-LDL/LOX-1 in endplate chondrocyte senescence and calcification

**DOI:** 10.1186/s10020-024-00887-8

**Published:** 2024-08-09

**Authors:** Tan Bing, Xiang Shanlin, Wang Jisheng, Hao Jie, Cao Ruichao, Zhang Zhiwei, Yu Bin, Ma Zhaoxin, Hu Zhenming, Zhou Nian

**Affiliations:** 1 Department of Spine Surgery, The Third Hospital of Mian Yang, Sichuan Mental Health Center, 621000, Mianyang, People’s Republic of China; 2 Department of Orthopedics, Orthopedic Laboratory of Chongqing Medical University, The First Affiliated Hospital of Chongqing Medical University, 400000, Chongqing, People’s Republic of China; 3 Department of Radiology, The First Affiliated Hospital of Chongqing Medical University, 400000, Chongqing, People’s Republic of China; 4 Department of Pharmacy, The Third Hospital of Mian Yang, Sichuan Mental Health Center, 621000, Mianyang, People’s Republic of China

**Keywords:** Intervertebral disc degeneration, Cell senescence and calcification, Lipid metabolism disorders, Oxidized low-density lipoprotein, Lectin-like oxidized low-density lipoprotein receptor 1

## Abstract

**Background:**

Lipid metabolism disorders are associated with degeneration of multiple tissues and organs, but the mechanism of crosstalk between lipid metabolism disorder and intervertebral disc degeneration (IDD) has not been fully elucidated. In this study we aim to investigate the regulatory mechanism of abnormal signal of lipid metabolism disorder on intervertebral disc endplate chondrocyte (EPC) senescence and calcification.

**Methods:**

Human intervertebral disc cartilage endplate tissue, cell model and rat hyperlipemia model were performed in this study. Histology and immunohistochemistry were used to human EPC tissue detection. TMT-labelled quantitative proteomics was used to detect differential proteins, and MRI, micro-CT, safranin green staining and immunofluorescence were performed to observe the morphology and degeneration of rat tail intervertebral discs. Flow cytometry, senescence-associated β-galactosidase staining, alizarin red staining, alkaline phosphatase staining, DCFH-DA fluorescent probe, and western blot were performed to detect the expression of EPC cell senescence, senescence-associated secretory phenotype, calcification-related proteins and the activation of cell senescence-related signaling pathways.

**Results:**

Our study found that the highly expressed oxidized low-density lipoprotein (ox-LDL) and Lectin-like oxidized low-density lipoprotein receptor 1 (LOX-1) in human degenerative EPC was associated with hyperlipidemia (HLP). TMT-labelled quantitative proteomics revealed enriched pathways such as cell cycle regulation, endochondral bone morphogenesis and inflammation. The rat model revealed that HLP could induce ox-LDL, LOX-1, senescence and calcification markers high expression in EPC. Moreover, we demonstrated that ox-LDL-induced EPCs senescence and calcification were dependent on the LOX-1 receptor, and the ROS/P38-MAPK/NF-κB signaling pathway was implicated in the regulation of senescence induced by ox-LDL/LOX-1 in cell model.

**Conclusions:**

So our study revealed that ox-LDL/LOX-1-induced EPCs senescence and calcification through ROS/P38-MAPK/NF-κB signaling pathway, providing information on understanding the link between lipid metabolism disorders and IDD.

**Supplementary Information:**

The online version contains supplementary material available at 10.1186/s10020-024-00887-8.

## Introduction

The high prevalence of Low Back Pain (LBP) has positioned it as a leading cause of disability globally, impacting approximately 7.5% of the population in 2020, which poses a serious threat to both physical and mental health, significantly affecting the overall quality of life (GBD Low Back Pain Collaborators 2023). Intervertebral disc degeneration (IDD) is the primary cause of LBP. However, despite its prevalence and severity, there are no curative or effective disease-modifying treatments for IDD, largely due to a limited understanding of its pathogenesis. IDD can be attributed to various factors, such as biological stress, metabolic disorders, nutritional disorders, etc.; however, the specific mechanisms underlying the disease remain elucidated (Wu et al. 2020; Cheng et al. 2021). Emerging evidence suggests that dysregulation of pro-inflammatory adipokines and elevated concentrations of circulating lipid resulting from lipid metabolism disorders may increase the risk of IDD (Teraguchi et al. 2016; Yi et al. 2023; Ruiz-Fernández et al. 2019; Li et al. 2022a; Shi et al. 2020; Chen et al. 2021). Nevertheless, the relationship between lipid metabolism disorders and IDD has not been elucidated yet, leaving the role and potential molecular mechanism of lipid metabolism disorders in IDD still unclear.

The intervertebral disc (IVD) is composed of three main parts: the nucleus pulposus (NP), the annulus fibrosus (AF) and the cartilage end plates (CEP) (Sun et al. 2021). The NP, which acts as a hydrogel-like core, is crucial for maintaining disc structural stability and biomechanical balance (Zhou et al. 2022). The resident NP cells regulate the metabolic processes of the extracellular matrix (ECM) through producing proteoglycan and collagen (Sun et al. 2021). Senescence and apoptosis of NP cells, along with progressive degeneration of ECM, are primary pathological changes observed in IDD (Xin et al. 2022). The CEP serves as the main pathway for nutrient supply and metabolite exchange in the IVD (Wang et al. 2022). Research has demonstrated a significant and independent association between CEP degeneration and IDD, suggesting that CEP degeneration may be the initial factor contributing to IDD (Jiang et al. 2019). Degenerative CEPs exhibit evident senescence phenotypes, increasing the risk of IDD occurrence and development (Wang et al. 2016; Yin et al. 2021; Brown et al. 2017). Furthermore, CEP calcification impairs its function in nutrient transport, pressure buffering, and metabolite exchange within the IVD, accelerating the onset and progression of IDD (Zehra et al. 2022). Additionally, CEP in contact with blood vessels may be early perceivers or victims of lipid metabolism disorders (Francisco et al. 2022). However, the pathogenic role of lipid metabolism disorders in CEP degeneration has not been documented.

Oxidized low-density lipoprotein (ox-LDL) is a product of lipid peroxidation formed by the oxidative modification of low-density lipoprotein (LDL), which is a primary pathogenic factor in lipid metabolism disorders (Khatana et al. 2020; Ahmadi et al. 2021). The LOX-1 receptor, consisting of 273 amino acids, serves as the principal cell membrane receptor of ox-LDL. LOX-1 is classified as a type II transmembrane protein and belongs to the C-type lectin superfamily (Barreto et al. 2021; Akhmedov et al. 2021). Intriguingly, LOX-1 is also expressed in IVD cells, particularly in degenerative intervertebral EPCs (Li et al. 2017). A growing body of evidence has established that the binding between LOX-1 and ox-LDL can induce cellular damage and senescence, thereby promoting the onset and progression of age-related degenerative diseases (Khatana et al. 2020; Barreto et al. 2021; Li et al. 2017). The activation of oxidative stress and inflammation-related signaling pathways has also been demonstrated to induce senescence in endothelial cells and smooth muscle cells, stimulate senescence-associated secretory phenotype (SASP), and promote calcification, ultimately contributing to the development of atherosclerosis in lipid metabolism disorders (Grootaert et al. 2018; Ahmad and Leake 2019; Song et al. 2017). However, the relationship between the expression of ox-LDL/LOX-1 in IDD and lipid metabolism disorders, as well as their regulatory effects on the pathological features of EPCs, require further exploration and analysis.

In our study, we comprehensively analyzed the effects of lipid metabolism disorders, represented by hyperlipidemia (HLP), on IDD. We systematically confirmed that HLP induced CEP degeneration and upregulated the expression of ox-LDL/LOX-1. Additionally, we further revealed that ox-LDL/LOX-1 promoted senescence and calcification of CEPs, ultimately contributing to the development of IDD in the context of persistent lipid metabolism disorder. Notably, the ROS/P38-MAPK/NF-κB pathway was involved in ox-LDL/LOX-1-induced EPCs senescence and calcification, suggesting a novel therapeutic approach for IDD.

## Methods

### Patient samples

The CEP specimens used in this study were obtained from patients with lumbar disc herniation who were diagnosed and treated at the First Affiliated Hospital of Chongqing Medical University between September 2021 and September 2022. The cohort consisted of 25 males and 8 females, with an average age of 39.5 ± 6.9 years. The degree of IDD and CEP degeneration was assessed by three experienced spinal surgeons using the modified Pfirrmann and Total End Plate Damage Score (TEPS) (Hollenberg et al. 2021; Rajasekaran et al. 2008) grading system through magnetic resonance imaging (MRI). The above specimens were categorized into three groups: the lumbar fracture group (LVF, n = 7), the intervertebral disc degeneration group (IDD, n = 16), and the hyperlipidemia combined with intervertebral disc degeneration group (HLP (IDD), n = 10). The IDD degree of the LVF group ranged from Pfirrmann I to II, while the IDD group ranged from III to VI, and the HLP (IDD) group ranged from III to VII. The corresponding sample data was presented in Supplementary Table 1. This study was ethically approved by the Ethics Committee of the First Affiliated Hospital of Chongqing Medical University (No.2021–251), and informed consent was obtained from the donors. The research was conducted in accordance with the ethical norms outlined by the World Medical Association as stated in the Declaration of Helsinki.

### Animals and reagents

All animal experiments were approved by the Animal Use and Ethics Committee of Chongqing Medical University (No.2021-251). In this study, a total of 30 male SD rats weighing between 210 and 297 g, were randomly assigned to either the normal diet group (control group, n = 15) or the high-fat diet group (HFD group, n = 15). The control group received standard pellet feed, while the HFD group was provided with a high-fat diet through daily quantitative feeding. The feed was supplied by Jiangsu Collaborative Pharmaceutical Bioengineering Co., Ltd. The blood lipid was measured at 2 months, and the hyperlipidemia rat model was successfully constructed according to the blood lipid level. After feeding for 3 months, the blood lipid level of the rats was measured. According to the blood lipid level, it was determined that the rats maintained a stable high blood lipid level. The rat model of intervertebral disc degeneration was successfully constructed by immunohistochemistry, micro-CT and MRI.

For the immunohistochemical experiments, three rats from each group were selected after 3 months and 6 months of feeding, respectively. After excluding rats with diabetes, the number of rats analyzed for serum content at 6-month was 9 in the control group and 7 in the HFD group.

### Cell culture and transfection

Human endplate chondrocytes (EPCs) were isolated and cultured as described previously (Zhou et al. 2016). The EPCs were initially cultured in serum-free DMEM medium (Gibco, USA) for 24 h. Subsequently, the cells were randomly divided into three groups: control, n-LDL (50 μg/mL), and ox-LDL (50 μg/mL), and treated for 24 h. Ox-LDL,n-LDL was purchased from Yiyuan Biotech (Guangzhou, China).Prior to the treatment, EPCs were pretreated with the reactive oxygen species (ROS) inhibitor NAC (Beyotime, China)/p38 inhibitor SB203580 (Sigma, USA)/NF-κB inhibitor QNZ (MCE, USA) for 1 h, followed by stimulation with ox-LDL (50 μg/mL) for 24 h. For gene silencing, EPCs were transfected with siRNA duplex targeting human OLR1 (LOX-1) and negative control (NC) siRNA, both designed and synthesized by Tsingke Biotechnology, China (Supplementary Table 2). The siRNAs were introduced into the cells using Endoectin^™^-Max (GeneCopoeia, EF003) following the manufacturer’s instructions. After 24 h of transfection, the cells were washed with phosphate buffered saline (PBS) and the medium was replaced with complete medium. The cells were then treated with ox-LDL (50 μg/mL) for 24 h. Finally, the cells were collected for qRT-PCR or western blotting to evaluate the knockdown effect.

### Cell viability assay

The CCK-8 assay was performed using the CCK-8 kit (MCE, USA). Initially, EPCs were seeded in 96-well plates at a density of 5 × 10^3^ cells per well and then incubated with various concentrations of ox-LDL for 24 h. Following this incubation, the cells were washed with PBS, and 100 μL DMEM/F-12 (including 10 μL CCK-8 solution) was added to each well. The plates were then incubated at 37 ℃ for 2 h. Finally, the absorbance of each well was measured by using a microplate reader at a wavelength of 450 nm.

### Flow cytometry

Cells were seeded in 12-well plates and incubated at 37 ℃, 5% CO_2_ incubator for 24 h. After exposure to ox-LDL for 24 h, the cells were digested and centrifuged by trypsin, resuspended in PBS, and centrifuged again. The cells were suspended and fixed with 70% ethanol at 4 ℃ overnight. After fixation, cells were washed with PBS and stained with 300 µL propidium iodide/RNase staining buffer (BD Pharmigen) at 4 ℃in the dark for at least 15 min. Stained cells were immediately analyzed for propidium iodide fluorescence using BD FACSCanto II. Cell cycle analysis was performed using the Cell Cycle platform in Flow Jo v10. Model fittings were done with either the Watson Pragmatic algorithm or Dean-Jett-Fox algorithm with unconstrained or constrained settings (G1 × 2), minimizing the root mean square error (Zhou et al. 2016).

### Western blot (WB)

Protein preparation and immunoblotting methods were performed as previously described (Hollenberg et al. 2021). The antibodies used in this article were listed below: anti-ox-LDL(1:400,PU224155,Abmart,China),anti-Aggrecan (1:1000, ab3773, Abcam, UK), anti-MMP13 (1:1000, #69926S, Cell Signaling Technology, USA), anti-LOX-1 (1:500, TD6522, Abmart, China), anti-p16INK4a (1:1000, ab270058, Abcam, UK), anti-p21 (1:5000, ab109520, Abcam, UK), anti-p-p38 (1:1000, ab4822, Abcam, UK), anti-p38 (1:2000, ab170099, Abcam, UK), anti-p-p65 (1:1000, ab76302, Abcam, UK), anti-p65 (1:2000, ab16502, Abcam, UK), anti-Collagen II (COL2, 1:2000, ab34712, Abcam, UK), anti-Collagen I (COL1, 1:1000, ab138492, Abcam, UK), anti-MMP3 (1:5000, ab34712, Abcam, UK), anti-MMP9 (1:5000, ab34712, Abcam, UK), anti-IL-1β (1:1000, ab254360, Abcam, UK), anti-IL-6 (1:1000, ab233706, Abcam, UK), goat anti-mouse IgG H&L (1:5000, ab205719, Abcam, UK), goat anti-rabbit IgG H&L (1:5000, ab6721, Abcam, UK).

### TMT-labeled quantitative proteomics

The TMT-labeled quantitative proteomics experiment was performed according to previously described methods (Zheng et al. 2020). Rat IVD specimens were collected and sent to LC-Bio Technology for subsequent TMT proteomic analysis. The quantitative proteomic analysis consisted of six steps: sample preparation, SDS-PAGE separation, filter-assisted sample preparation (FASP) digestion, TMT labeling, reversed-phase peptide separation, and mass spectrometry. The MS/MS raw files were processed using the MASCOT engine (Matrix Science, UK) embedded into Proteome Discoverer 2.2 and searched against protein databases for peptide identification. A reverse database search strategy was employed with a 1% peptide and protein false discovery rate threshold. The differentially expressed proteins for Gene Ontology (GO) and COG/KOG analysis were defined as those with a fold change > 1.2 and a P value (Student’s t test) < 0.05.

### Histological and immunohistochemical analyses

For histological analysis, human and rat IVD specimens were fixed in 4% formaldehyde for 24 h, decalcified in 10% EDTA for 21 days, dehydrated by a gradient ethanol serious, and subsequently embedded in paraffin. The specimens were sliced longitudinally into 5 μm thick sections and stained with hematoxylin–eosin (HE) and safranin-fast green (SO).

HE staining was performed using a HE Staining Kit (C0105M, Beyotime, China). The paraffin-embedded sections were dewaxed twice with fresh xylene for 5–10 min each time, followed by dehydration using a gradient of alcohol (100%, 90%, 80%, 70%) for 2 min. Subsequently, the sections were dehydrated with distilled water for 2 min. Then, the treated slices were incubated with hematoxylin dye at room temperature for 5–10 min, and washed with distilled water to remove excess dye. After eosin staining for 2 min, the sections were dehydrated with gradient alcohol (70%, 80%, 90%, 100%) for 10 s each. The next step involved transparentizing the sections in fresh xylene for 10 min before sealing with neutral balsam. Images of sections were obtained through optical microscopy. The experimental procedure of SO staining was similar to HE staining, except that after HE, eosin staining was replaced by applying solid green dyeing solution and saffron O dyeing solution for 5 min each, without intermediate washing—just removing excess dye.

For immunohistochemical staining, the sections were incubated in 0.3% hydrogen peroxide for 20 min, and antigen retrieval was performed at 65 ℃ in 0.01 mol/L citrate buffer (pH 6.0) for 20 min. Following blocking with 5% goat serum for 1 h, the sections were incubated with primary antibody at 4 ℃ overnight. The next day, the sections were incubated with HRP-conjugated secondary antibodies (1:1000, 111-035-003, Jackson ImmunoResearch, UK) at room temperature for 1 h. A HRP-streptavidin system (Dako, Denmark) was then utilized to detect positive areas, followed by counterstaining with hematoxylin.

### Micro-computed tomography (Micro-CT) and MRI assays

As described previously, micro-CT and analysis were performed (Lyu et al. 2021). The micro-CT machine (Bruker Skyscan 1176, Belgium) was used for the detections. Each sample was scanned with the following parameters: 65 kV source voltage, 381 μA current, 1 mm aluminum filter, 0.5° rotation step, 18 μm image pixel size, 500 ms exposure time, 360° scan, and four frames per rotation. Three-dimensional (3D) reconstructions were conducted using NRecon software (Bruker MicroCT, Kontich, Belgium), and bone volume (BV) was calculated with Magics software. MRI was utilized to determine IDD. All structural MRI scans were acquired using a Philips Intera Achieva 3.0 T MRI scanner (Philips Medical Systems B.V.). MRI was conducted using the following parameters:1 mm slice thickness; 256 × 256 matrix; echo time (TE) = 2 ms; repetition time (TR) = 1200 ms; flip angle = 90°. The degree of IVD in MRI images were calculated and analyzed using FrameLink software version 1.0 (Medtronic).

### Immunofluorescence

The EPCs slides were initially treated with 4% paraformaldehyde at room temperature for 15 min. Then, the cell slides were permeabilized with 0.3% Triton X-100 (MP Biomedical, USA) for another 15 min. After blocking with QuickBlock^™^ immunostaining blocking solution (Beyotime, China) for 1 h, the cell slides were stained with anti-MMP13 (1:100, ab39012, Abcam, UK), anti-p16INK4a (1:50, ab270058, Abcam, UK) and anti-Collagen II (COL2, 1:100, ab34712, Abcam, UK) antibodies, and incubated overnight at 4 ℃. The next day, the slides were incubated with the corresponding Cy 3-conjugated secondary antibody (1:200, Abnova, China) in the dark at room temperature for 2 h. Finally, the slides were stained with 4’,6-diamidino-2-phenylindole (DAPI, Beyotime, China) in the dark at room temperature for 20 min. After each step, the slides were washed three times with PBS for 5 min each time. The cell slides were then observed under a fluorescence microscope (BX53, Olympus, Japan) or a fluorescence confocal microscope (Nikon, Japan), and the fluorescence intensity was quantified using ImageJ software.

### Biochemical analysis

Rats were rapidly anesthetized by intraperitoneal injection of 3% pentobarbital sodium (20 mg/kg). After administering anesthesia, 1.5 mL of blood was collected from the posterior orbital venous plexus using a capillary glass tube. The collected blood was subsequently incubated at room temperature for 1 h and then centrifuged at 3000 rpm for 10 min. The resulting sample was immediately transferred into a 1.5 mL EP tube. The levels of total cholesterol (TC), triglycerides (TG), low-density lipoprotein cholesterol (LDL-C), high-density lipoprotein cholesterol (HDL-C), and fasting blood glucose (FBG) were measured using an automatic biochemical analyzer (BS360S, Mindray).

### Detection of reactive oxygen species

The EPCs were inoculated in 24-well plates and divided into groups based on different interventions. After discarding the medium, the plates were washed twice with basic medium. Then 200 μL DCFH-DA (Beyotime, China) was added to each well, and the plates were incubated at 37 ℃ for 20 min in the dark. After washing twice with PBS, the fluorescence intensity of ROS was observed under an inverted fluorescence microscope.

### Alkaline phosphatase staining and alizarin red staining

EPCs were seeded in 12-well plates at a density of 5 × 10^5^ cells per well. Upon reaching 80% confluence, the cells were cultured in osteogenic induction medium (Saiye Biotechnology, China), with the medium replaced every 2 days, and treated with ox-LDL (50 μg/mL). ALP staining and activity determination were performed 7 days after osteogenic induction according to the manufacturer’s instructions (Beyotime, China). After 21 days of osteogenic induction, alizarin red S staining (Biotechnology Co, China) was performed to evaluate mineral deposition.

### The senescence-associated beta-galactosidase (SA-β-gal) staining assay

After ox-LDL intervention, EPCs in the 6-well plate were washed twice with PBS. Subsequently, 1 mL SA-β-Gal staining fixative (Beyotime, China) was added to each well and fixed the cells at room temperature for 15 min. Following that, the wells were washed three times with PBS. Finally, 1 mL prepared staining working solution (Beyotime, China) was added to each well, and the plates were incubated overnight in a 37 ℃, CO_2_-free incubator. The images were captured using a high-resolution microscope and statistically analyzed.

### Quantitative real-time polymerase chain reaction (qRT-PCR) analysis

The total RNA was extracted using RNA purification kit (Takara, Japan), and cDNA synthesis was performed on 500 ng of total RNA using the reverse transcription kit (MCE, USA). Subsequently, qRT-PCR analysis was conducted using the SYBR Green qPCR premix (MCE, USA). Relative gene expression levels were determined utilizing the 2^−ΔΔCT^ method, with β-actin serving as the internal reference. Primers for amplification were obtained from Takara and their primer sequences were as follows:

LOX-1-F:5′-AGCCTGATGAGAAGTCAAATGG-3′ and.

LOX-1-R:5′-TCGGACTACTCTTCAGTTTACC-3′;

P21-F:5′-CCGCCCCCTCCTCTAGCTGT-3′ and.

P21-R: 5′-CCCCCATCATATACCCCTAACAC-3′;

Runx2-F:5′-CCATAACCGTCTTCACAAATCC-3′and.

Runx2-R:5′-GGTATTGGCAGAAGTGTTTAGG-3′;

β-actin-F: 5′-CTCCATCCTGGCCTCGCTGT-3′ and.

β-actin-R: 5′-GCTGTCACCTTCACCGTTCC-3′.

### Statistical analysis

The statistical analysis was employed using GraphPad Prism 8 software (GraphPad Software, USA). The data were exhibited as mean ± standard deviation (SD). Statistical differences between two groups were analyzed using Student’s t-test, while one-way analysis of variance (ANOVA) followed by Tukey’s post hoc test was used to compare differences among multiple groups. Statistical significance was defined as *p* < 0.05, with non-significance (ns) being indicated as *p* > 0.05. Statistical significance levels were denoted by asterisks: *p < 0.05, **p < 0.01, ***p < 0.001.

## Results

### ox-LDL, LOX-1 receptor and cell senescence- and calcification-related proteins are upregulated in degenerative CEP tissues, influenced by lipid metabolism disorders

**Fig. 1:**
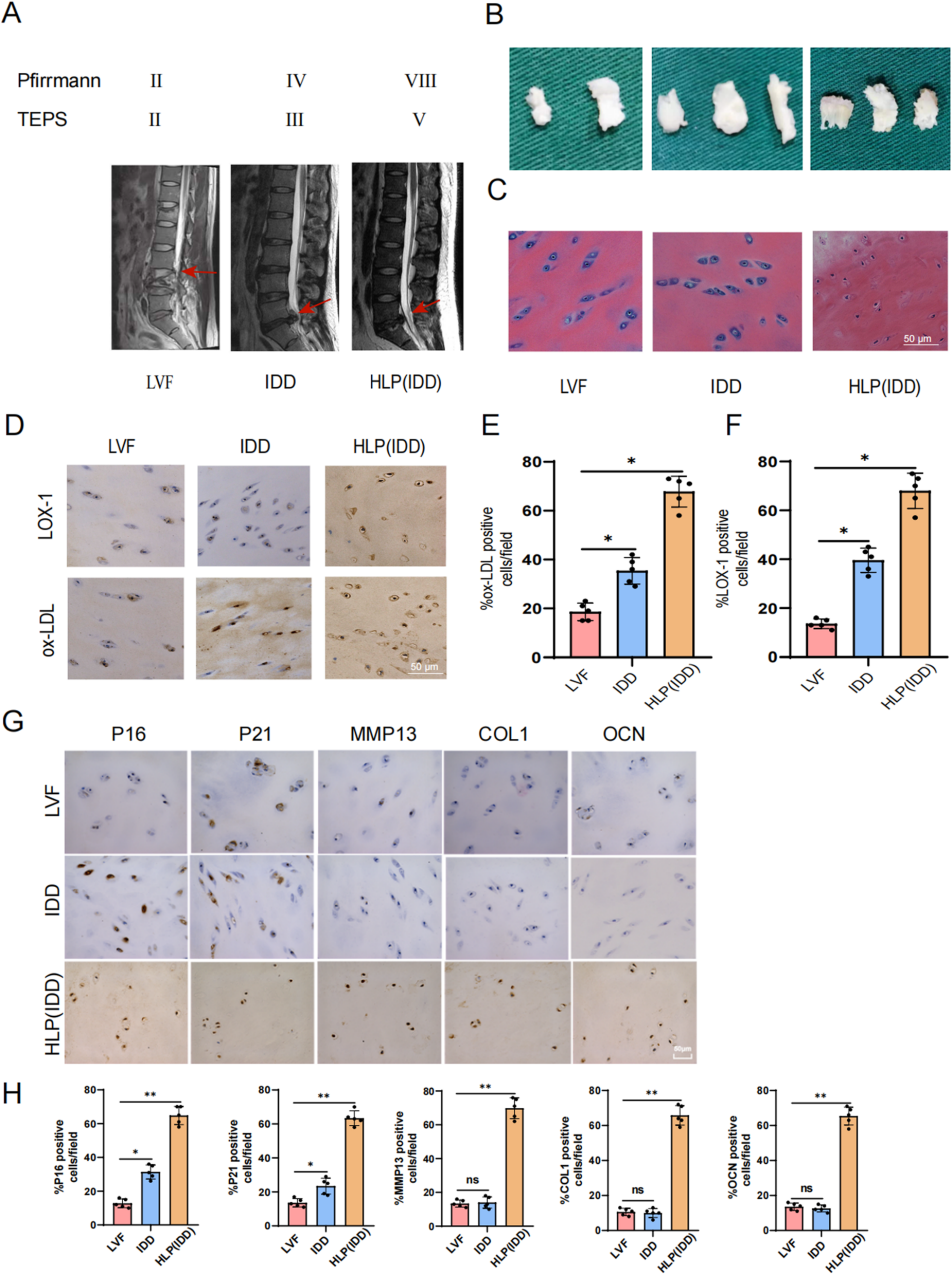
The expression of ox-LDL and LOX-1 in degenerative cartilage endplate tissues. **A** MRI was used to examine human intervertebral disc, with T2-weighted signal, and representative MRI images were displayed. Each group consisted of 5 samples, with the corresponding intervertebral disc indicated by red arrows. **B** General organization of human cartilage end plates. **C** H&E staining of human CEP tissues. Scale bar = 50 μm. **D** Immunohistochemical staining of ox-LDL, LOX-1 in CEP tissues. Scale bar = 50 μm. **E**, **F** Quantitative analysis of ox-LDL, LOX-1 expression in human CEP tissues. **G** Immunohistochemical staining of cell senescence and calcification markers P16, P21, MMP13, COL1, OCN in human CEP tissues. Scale bar = 50 μm. **H** Quantitative analysis of P16, P21, MMP13, COL1, OCN expression in human CEP tissues. Each image represented a sample from a total of 5 examined samples, Scale bar = 500 μm. Compared to LVF group, *p < 0.05, **p < 0.01, ns indicated non-significance difference

The expression of oxidized low density lipoprotein (ox-LDL) and Lectin-like oxidized low-density lipoprotein receptor 1 (LOX-1) in cartilage endplate (CEP) tissues of degenerative Intervertebral Disc(IVD) has been previously demonstrated to be higher compared to that in NP(Nucleus Pulposus) and AF(Anulus Fibrosus) (Li et al. 2017). In order to further investigate the association between ox-LDL/LOX-1 and IVD, we collected CEP tissues from Lumbar vertebral fracture (LVF), Intervertebral Disc Degeneration(IDD), and Hyperlipidemia with intervertebral disc degeneration (HLP (IDD)) patients for analysis. MRI scans showed that the severity of IVD grade and CEP degeneration was more pronounced in the HLP (IDD) group compared to the IDD and LVF groups, with relatively mild degeneration observed in the LVF group **(**Fig. 1A**)**. Additionally, upon examination of several CEP tissues from each group, it was observed that those from the LVF group appeared white and transparent with a relatively small size; those from the IDD group were significantly thicker than those from the LVF group but exhibited a mild degenerative phenotype; while those from the HLP (IDD) group were relatively thick, yellow in color, and displayed characteristics of calcification **(**Fig. 1B**)**. Furthermore, cellular morphology was assessed through H&E staining, which revealed that cells in the IDD group appeared relatively flat and spindle-shaped with reduced extracellular matrix content along with enlarged cell nuclei compared to the cells in the LVF group. Interestingly, cells in the HLP (IDD) group showed significant shrinkage, indicating potential fibrosis and calcification **(**Fig. 1C**)**. Immunohistochemical experiments revealed significantly increased expression levels of ox-LDL and LOX-1 within the IDD group compared to the LVF group. Moreover, these protein expressions were even more pronounced within samples obtained from individuals in the HLP (IDD) category **(**Fig. 1D–F**)**. In addition, we employed immunohistochemistry to assess the expression of senescence- and calcification-related proteins in CEP tissues obtained from different clinical patients. As depicted in Fig. 1G, compared to the LVF group, the expression of P16 and P21 in CEP tissues from the IDD group were significantly upregulated, while the expression of MMP13, COL1, and OCN showed no significant difference. In the HLP (IDD) group, the expression levels of all proteins were significantly elevated (Fig. 1H), indicating a substantial increase in cell senescence and calcification. Overall, these results suggested that ox-LDL, LOX-1 and cell senescence- and calcification-related proteins were highly expressed within CEP tissues associated with IVD pathology, and highlighted that HLP may contribute to promoting their expression.

**Fig. 2:**
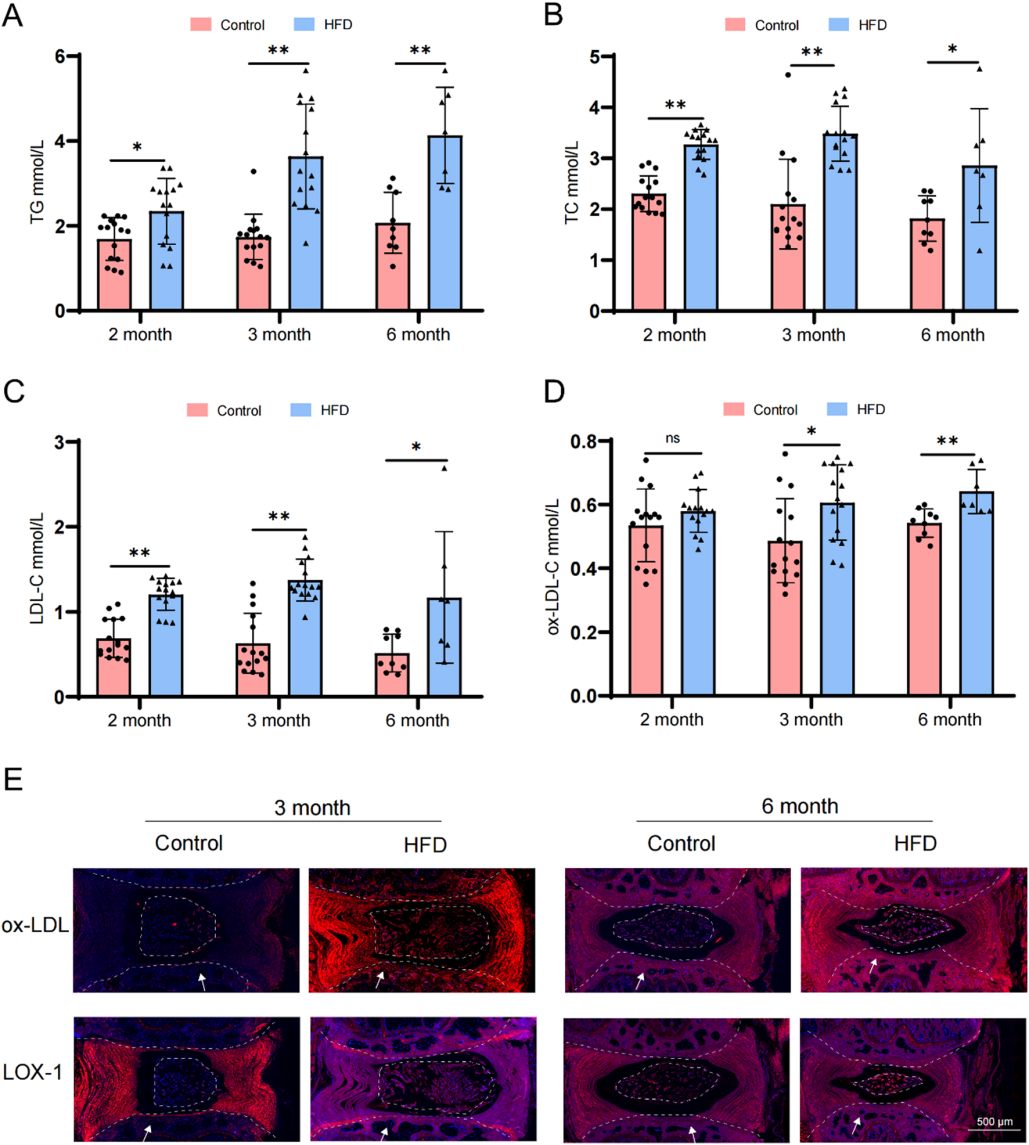
Detect the expression levels of ox-LDL and LOX-1 in cartilage endplate tissues from the HFD (High Fat Diet) group in animal model. **A**-**C** Detection of serum lipid levels in HFD-induced model of tail disc degeneration. **D** Serum ox-LDL levels were measured by ELISA. **E** Immunofluorescence representative images of ox-LDL, LOX-1. Each image represented a sample from a total of 5 examined samples, Scale bar = 500 μm. The white arrow indicated the location on the cartilage end plates. Compared to control group, *p < 0.05, **p < 0.01, ns indicated non-significance difference

To further validate our hypothesis, we utilized SD rats to construct a HFD-induced model of tail disc degeneration. Initially, we assessed the expression levels of TG, TC, and LDL-C in the serum of rats at different time points (30 rats in 2 months, 30 rats in 3 months, and 16 rats in 6 months). Remarkably elevated concentrations of TG, TC, and LDL-C were observed in the HFD-treated group compared to the control group at each time point **(**Fig. 2A–C**)**. This finding substantiated the successful construction of our animal model. Subsequently, we evaluated the expression of ox-LDL-C, and noted no significant difference after 2 months. However, at 3 and 6 months there was a substantial increase in ox-LDL-C concentration within the serum of the HFD group **(**Fig. 2D**)**. Fluorescence microscopy images further showed pronounced expression levels of both ox-LDL and LOX-1 in CEP tissues from the HFD group **(**Fig. 2E**)**, consistent with the ELISA results. Consequently, we concluded that ox-LDL and LOX-1 were highly expressed in the CEP tissues of IVD, highlighting that HLP significantly promoted their expression.

### Lipid metabolism disorders can induce cell senescence and calcification in cartilage endplate (CEP) tissues

**Fig. 3:**
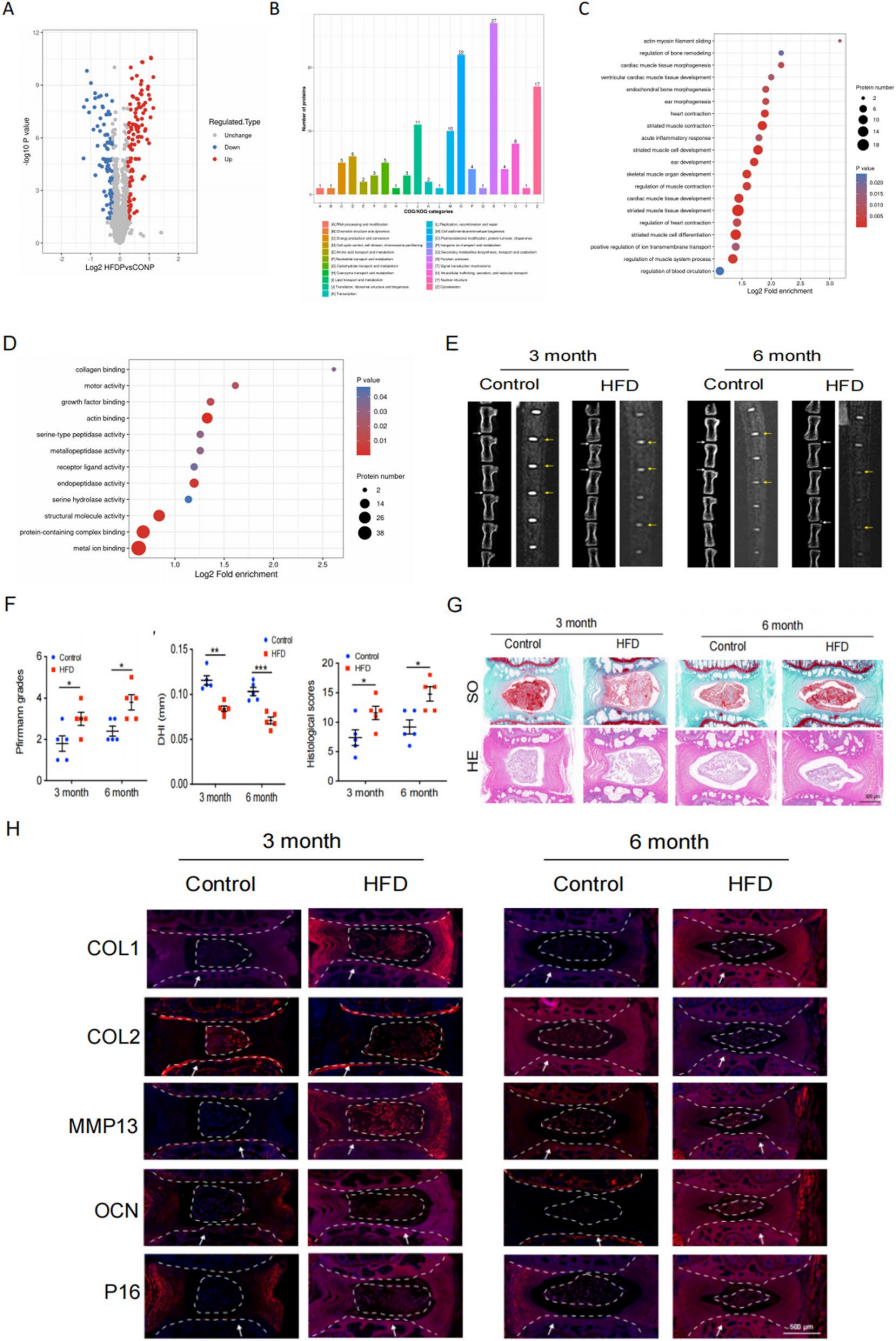
HFD (High Fat Diet) induces cartilage endplate cell senescence and calcification in rat intervertebral discs. **A** Differentially expressed protein volcano maps from quantitative proteomics. **B** COG /KOG functional classification. **C**, **D** GO enrichment analysis of differentially expressed proteins (biological processes and cellular components) derived from quantitative proteomics analysis. **E** Representative micro-CT and MRI images of caudal vertebrae from rats fed with HFD for 3 and 6 months (n = 5). White and yellow arrows indicate the target disc. **F** Statistical plot of Pfirrmann grade, DHI and histological scores (n = 5). **G** Representative images of HE, SO staining of the rat coccygeal spine IVD (n = 5). Scale bar = 500 μm. **H** Immunofluorescence representative images of MMP13, COL2, P16, OCN and COL1 (n = 5). Scale bar = 500 μm. White arrow: cartilage endplate. Compared to control group, *p < 0.05, **p < 0.01, ***p < 0.001

To elucidate the regulatory mechanism of lipid metabolism in intervertebral disc degeneration (IDD) we conducted a proteomic analysis on the Intervertebral Disc(IVD) tissues from both the HFD group and the control group. This analysis revealed 207 differentially expressed proteins in the HFD group, including 110 up-regulated proteins and 97 down-regulated proteins **(**Fig. 3A**)**. Subsequently, COG/KOG functional classification and GO analysis demonstrated that these differentially expressed proteins were enriched in pathways such as cell cycle regulation, chondrocyte development and calcification **(**Fig. 3B–D**)**. These results were consistent with those obtained from human tissue specimens. Based on these findings, we hypothesized that dysregulation of lipid metabolism may impact the processes of cell calcification and senescence.

Considering that previous experiments demonstrated significant differences in ox-LDL-C concentration at 3 and 6 months, we selected CEP tissues from rats at these time points for further investigation. CT and MRI images, along with statistical analyses, revealed a significant decrease in IVD signal intensity, reduced IVD height, and notably increased degeneration grade within the HFD group compared to the control group. Moreover, there was a marked reduction in the intervertebral disc height index (DHI). At 6 months, visible IVD calcification occurred (Fig. 3E), suggesting that HFD could induce IDD and calcification in rats. Additionally, histological staining and scoring were performed on IVD specimens obtained from the aforementioned animal models. As depicted in Fig. 3G, HE staining revealed a conspicuous presence of numerous star-shaped cells within the IVD tissues of the control group, exhibiting well-organized fibrous ring tissues. Conversely, in the HFD group, there was a collapse in intervertebral DHI and damage to the fibrous ring layer, which was also observed in SO staining. The Pfirrmann score was used to evaluate the degree of intervertebral disc degeneration. The Pfirrmann score of the intervertebral disc in the HFD group was significantly higher than that in the control group, indicating that the degree of intervertebral disc degeneration in the HFD group was significantly heavier than that in the control group(Fig. 3F).The intervertebral DHI in the HFD group was significantly higher than that in the control group, indicating that the degree of intervertebral disc degeneration in the HFD group was significantly heavier than that in the control group **( **Fig. 3F**).**The Thompson score of the intervertebral disc in the HFD group was significantly higher than that in the control group, indicating that the degree of intervertebral disc degeneration in the HFD group was significantly heavier than that in the control group **( **Fig. 3F**)**. Subsequently, the Thompson classification system was utilized to assess the histopathological score of the tissue. The statistical findings confirmed that HFD treatment could induce IVD degeneration in rats. (Fig. 3F). To further confirm the impact of HFD on rat senescence and tissue calcification, immunofluorescence experiments were conducted. Fluorescence microscopy images showed that P16, MMP13, OCN, and COL1 exhibited high expression levels in CEP tissues from the HFD group, while COL2 expression decreased significantly (Fig. 3H). This provided additional evidence supporting that HLP had an inducible effect on the calcification and senescence of EPCs.

### ox-LDL induced senescence and calcification of EPCs

**Fig. 4:**
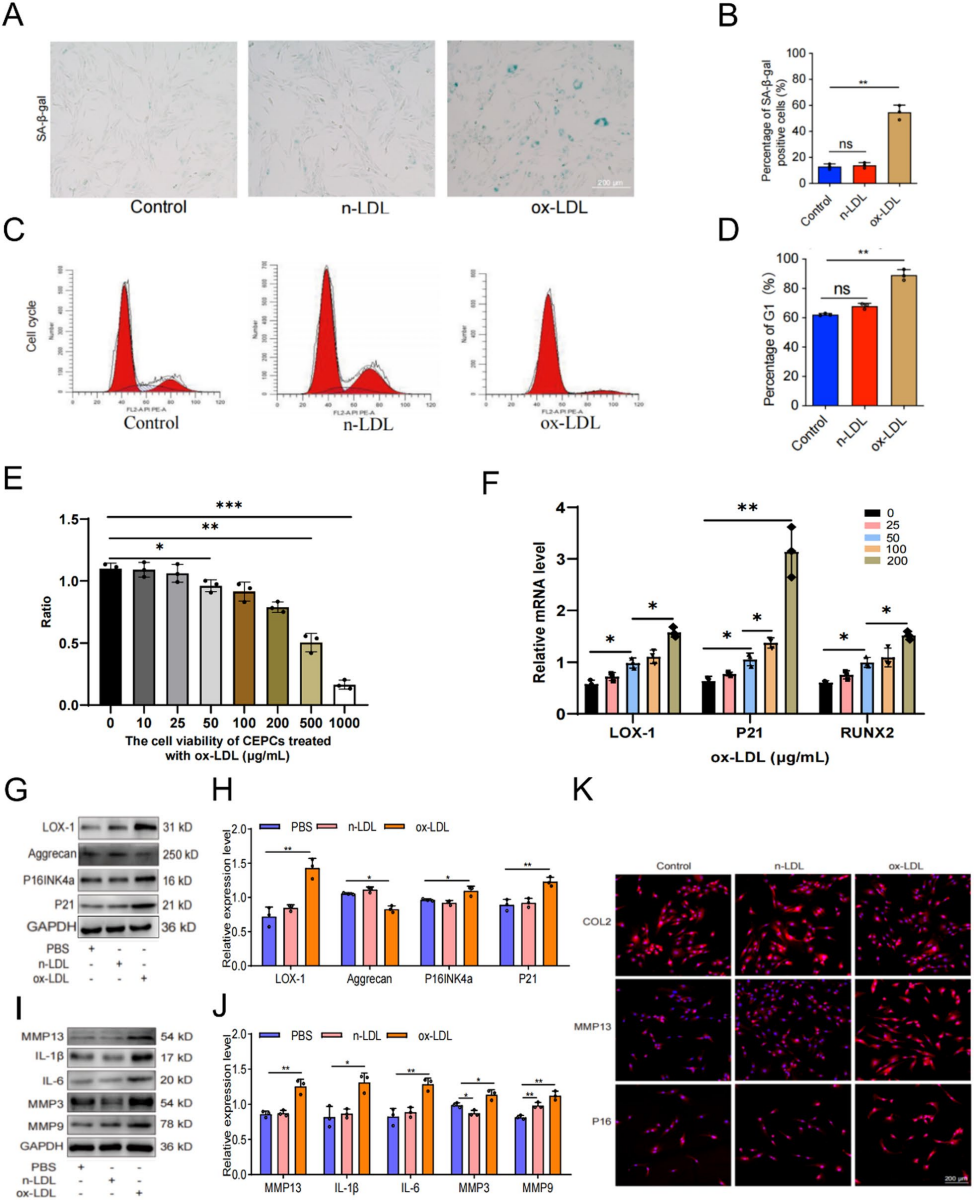
Ox-LDL induced endplate chondrocytes senescence. **A**,** B** Results of SA-β-Gal staining results and quantitative analysis (scale = 200 μm). **C**, **D** Cell cycle detection and analysis results. **E** The effects of different concentrations of ox-LDL on the cell viability of endplate chondrocytes. (F) The effects of various concentrations ox-LDL on mRNA expression levels of LOX-1, P21 and RUNX2. **G**-**J** Western blotting and quantitative analysis of senescence-related proteins. **K** COL2, MMP13, P16 representative immunofluorescence staining results. Compared to control group, **p < 0.01, ns indicated non-significance difference; Compared to 0 μg/mL group, *p < 0.05, **p < 0.01, ***p < 0.001; Compared to PBS group, *p < 0.05, **p < 0.01

Previous findings have demonstrated a significant upregulation of ox-LDL in HLP-induced IVD, and a strong correlation between HLP and the calcification and senescence of EPCs. To elucidate the relationship between ox-LDL expression and EPC calcification and senescence, we designed the following experiments for verification. The results depicted in Figures 4A, B exhibited a significantly elevated proportion of SA-β-gal-positive cells in the EPCs subjected to ox-LDL treatment, compared to both control and n-LDL treatment groups. This indicated a substantial increase in senescent cell population. Furthermore, flow cytometry analysis revealed a noticeable increase in the percentage of G1 phase cells following ox-LDL treatment (Figures 4C, D), suggesting the induction of cell cycle arrest. Additionally, we treated EPCs with varying concentrations of ox-LDL to assess its impact on cell viability and the mRNA expression of relevant genes. The results obtained from CCK-8 assay revealed a concentration-dependent inhibition of EPC activity by ox-LDL, with significant suppression of cell viability observed after 24 hours of treatment with 50 μg/mL **(**Figure 4E**)**.

We subsequently employed qRT-PCR, WB, and cell immunofluorescence techniques to assess alterations in cellular aging-related indicators. The qRT-PCR results demonstrated a progressive increase in the expression levels of P21 and RUNX2 as the concentration of ox-LDL treatment increased, with the highest levels observed at 200 μg/mL treatment **(**Figure 4F**)**. This finding suggested that ox-LDL could upregulate the expression of genes associated with senescence and calcification. The WB analysis revealed a significant increase in the expression levels of cell cycle-related factors (P16, P21, etc.) and SASP markers (IL-6, MMP, etc.) in the ox-LDL treatment group of EPCs compared to those in the control and n-LDL treatment groups **(**Figures 4G–J**)**, further validating these findings at the protein level. It is worth noting that immunofluorescence results also indicated heightened expression levels of P16 and MMP13 in the EPCs treated with ox-LDL relative to the control and n-LDL groups, while the expression of COL2 was significantly lower than that observed in the control group (Figure 4K). These findings collectively indicated that ox-LDL could induce senescence in EPCs.

**Fig. 5:**
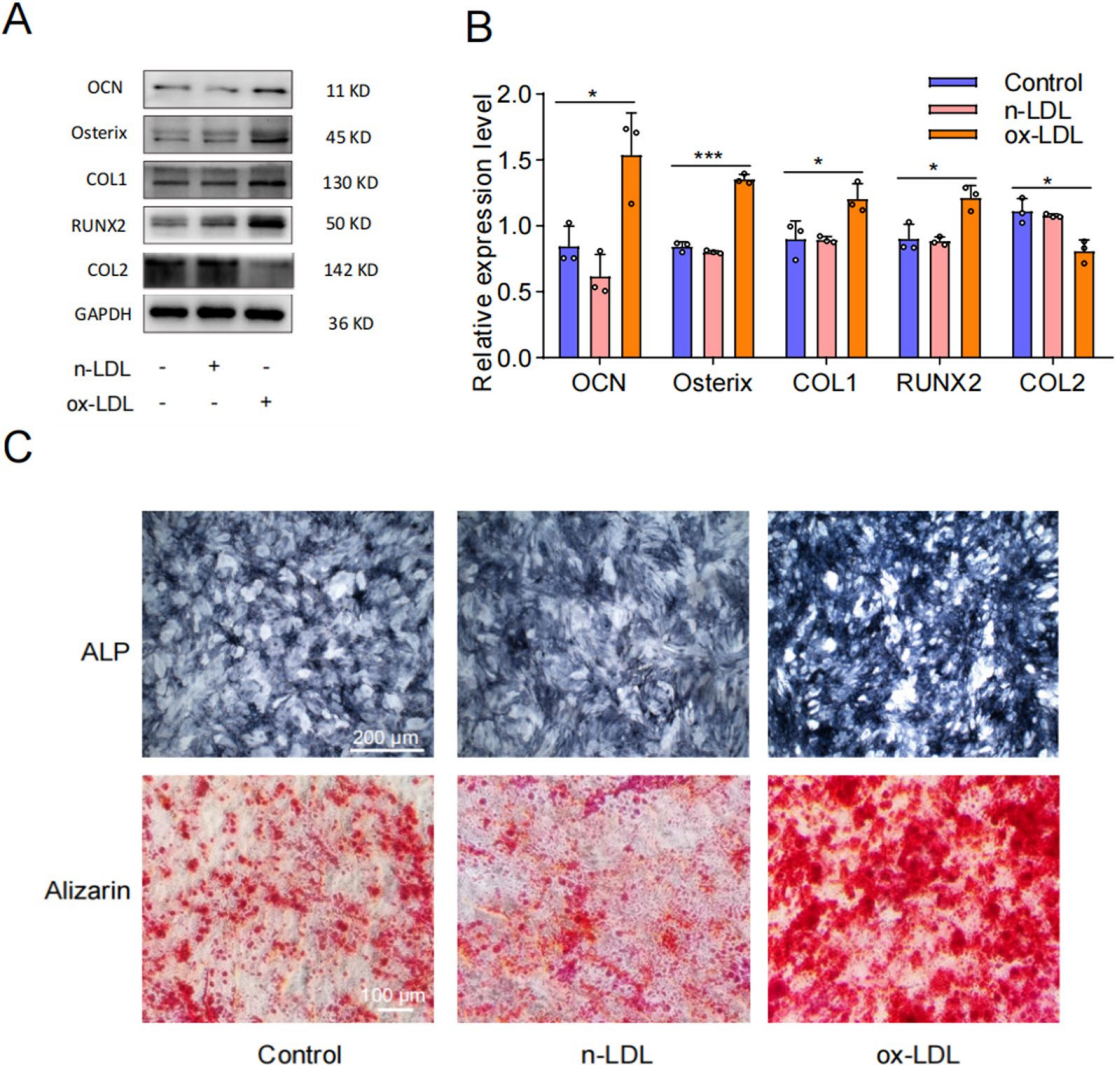
Ox-LDL induced calcification of endplate chondrocytes. **A**, **B** Western blotting and quantitative analysis of OCN, Osterix, COL1, RUNX2 and COL2. **C** Representative alkaline phosphatase and alizarin red staining results (n = 3). Compared to Control group, *p < 0.05, ***p < 0.001

To further investigate the impact of ox-LDL on EPC(endplate chondrocyte) calcification, we used WB and Alizarin Red/Alkaline Phosphatase (ALP) staining to detect relevant indicators. The WB results demonstrated that treatment with ox-LDL significantly upregulated the expression of OCN, Osterix, COL1, and RUNX2, while inhibiting the expression of COL2 (Fig. 5A, B). ALP staining revealed enhanced staining ability and activity following ox-LDL treatment, and Alizarin Red staining indicated that ox-LDL significantly enhanced the calcium nodule formation and staining intensity (Fig. 5C). In conclusion, our findings suggested that ox-LDL played a pivotal role in promoting cellular senescence and calcification in EPCs.

### The senescence and calcification of EPCs induced by ox-LDL depended on the LOX-1 receptor

**Fig. 6:**
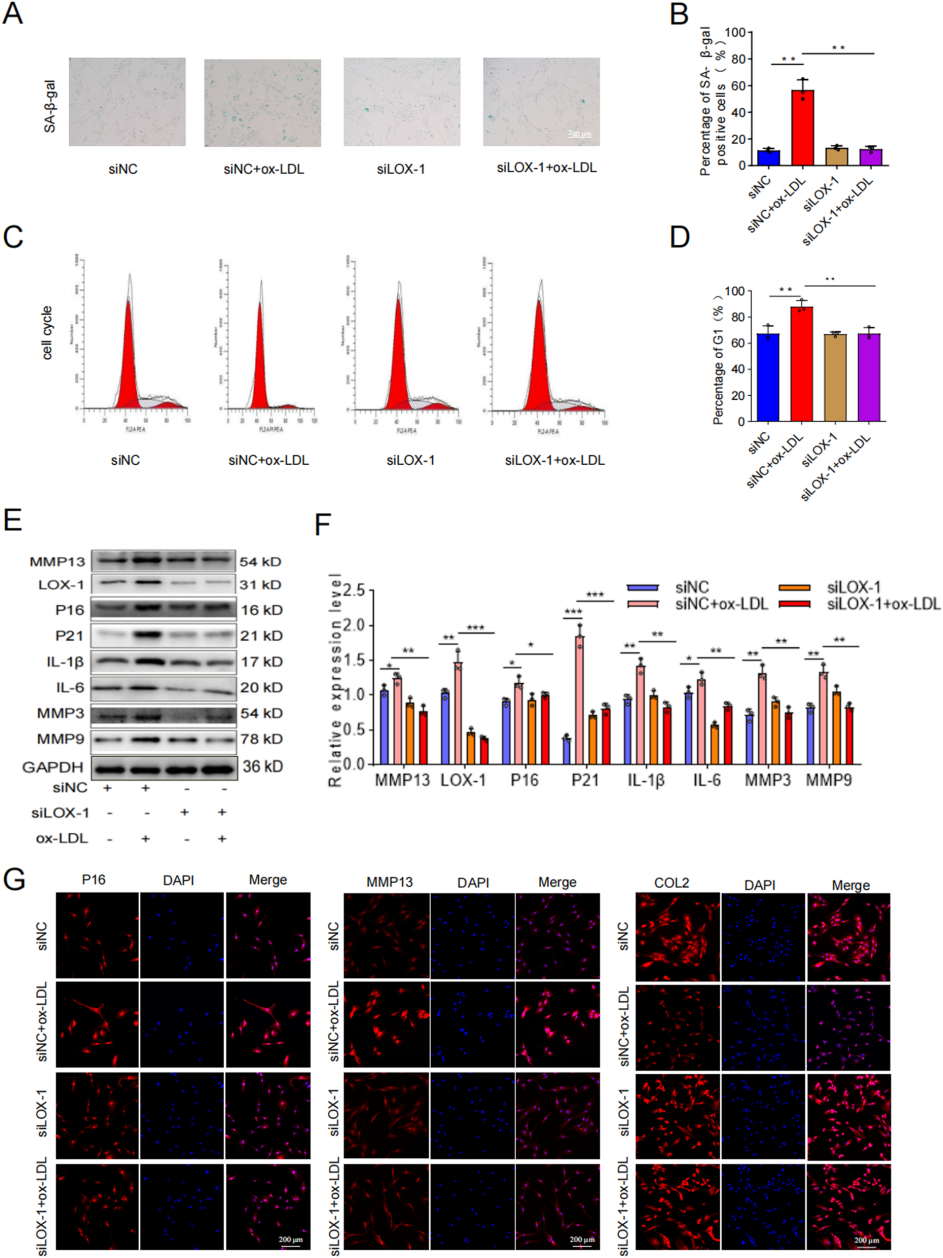
The induction of senescence in cartilage endplate cells by ox-LDL was mediated through LOX-1. **A**,** B** Results of SA-β-Gal staining and quantitative analysis (Scale bar = 200 μm). **C**,** D** Cell cycle detection and analysis results. **E**,** F** Western blotting and quantitative analysis of LOX-1, MMP13, p16INK4α, P21, IL-1β, IL-6, MMP3 and MMP9. **G**-**I** COL2, MMP13, P16 representative immunofluorescence staining results. Compared to siNC + ox-LDL group, *p < 0.05, **p < 0.01, ***p < 0.001

The binding of ox-LDL to LOX-1 and its impact on the development of various diseases have been previously demonstrated in studies. In order to determine whether the LOX-1 receptor mediates ox-LDL-induced senescence and calcification in EPCs, we analyzed the effects of ox-LDL treatment and siLOX-1/siNC treatment on senescence and calcification-related phenotypes in EPCs. Firstly, we observed that ox-LDL treatment could promote LOX-1 expression in EPCs (Fig. 4F–H). However, siLOX-1 treatment significantly inhibited LOX-1 expression in EPCs, and the addition of ox-LDL had no significant effect on LOX-1 expression (Fig. 6E, F). After confirming the regulatory relationship between ox-LDL and LOX-1 in EPCs, changes we further assessed changes in senescence-related markers using β-galactosidase staining, flow cytometry, WB, and cell immunofluorescence. The results revealed that ox-LDL treatment significantly increased the proportion of cells positive for senescence-related SA-β-Gal staining as well as G1 phase cells. However, these proportions were significantly reduced after siLOX-1 treatment and were comparable to those observed with siLOX-1 alone treatment group **(**Fig. 6A–D). Additionally, at the protein level, the overexpression of senescence-related proteins induced by ox-LDL treatment was restored to the level of the siLOX-1 alone treatment group after further treatment with siLOX-1 (Fig. 6E, F). This was also confirmed by immunofluorescence experiment (Fig. 6G–I), indicating that the addition of ox-LDL after inhibiting LOX-1 did not exert the original effect on the expression of senescence-related proteins. These findings suggested that LOX-1 mediated ox-LDL-induced EPCs senescence.

**Fig. 7:**
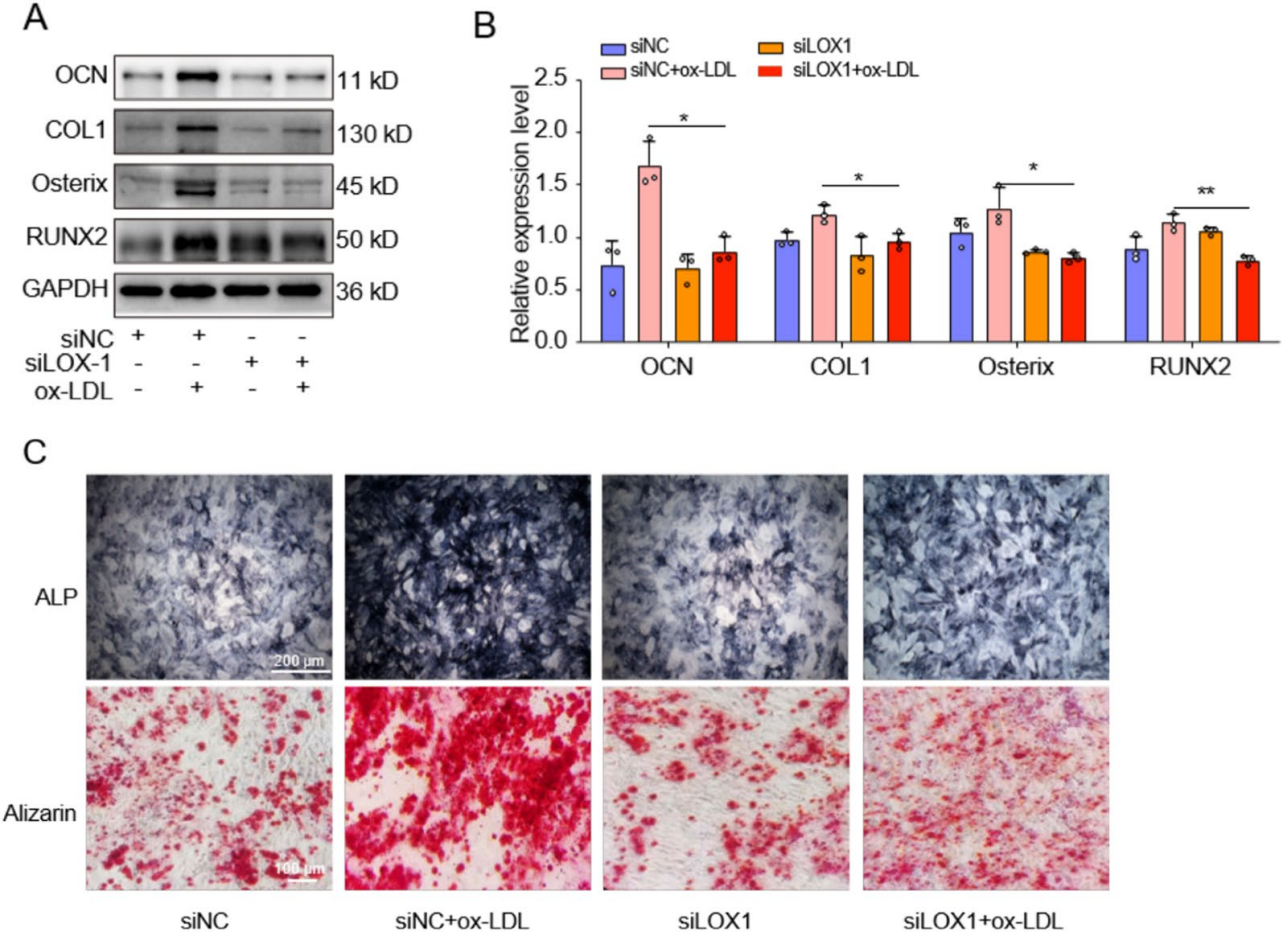
Ox-LDL induced calcification of cartilage endplate cells was mediated by LOX-1. **A**,** B** Western blotting and quantitative analysis of OCN, Osterix, COL1 and RUNX2 for calcification. **C** Representative alkaline phosphatase and alizarin red staining results (n = 3). Compared to siNC + ox-LDL group, *p < 0.05, **p < 0.01

To determine whether LOX-1 also mediated ox-LDL-induced EPCs calcification, we employed WB and ALP/Alizarin red staining to assess changes in markers associated with calcification. The WB analysis results revealed significantly decreased expression levels of calcification-related proteins in the ox-LDL + siLOX-1 group compared to the ox-LDL + siNC group, indicating that knockdown LOX-1 effectively inhibited ox-LDL-induced EPCs calcification (Fig. 7A, B). Furthermore, ALP staining demonstrated a higher number and more intense coloration of ALP-positive cells in the ox-LDL + siNC group as opposed to the ox-LDL + siLOX-1 group. Additionally, Alizarin red staining showed abundant formation of calcium nodules with darker staining intensity in the ox-LDL + siNC group, whereas only a small amount of Alizarin red staining was observed and no obvious calcium nodules were formed in the ox-LDL + siLOX-1 group (Fig. 7C). Therefore, our findings strongly suggested that LOX-1 played a crucial role in mediating ox-LDL-induced EPCs calcification.

### The P38-MAPK/NF-κB pathway was involved in ox-LDL/LOX-1-induced EPCs senescence and calcification

**Fig. 8:**
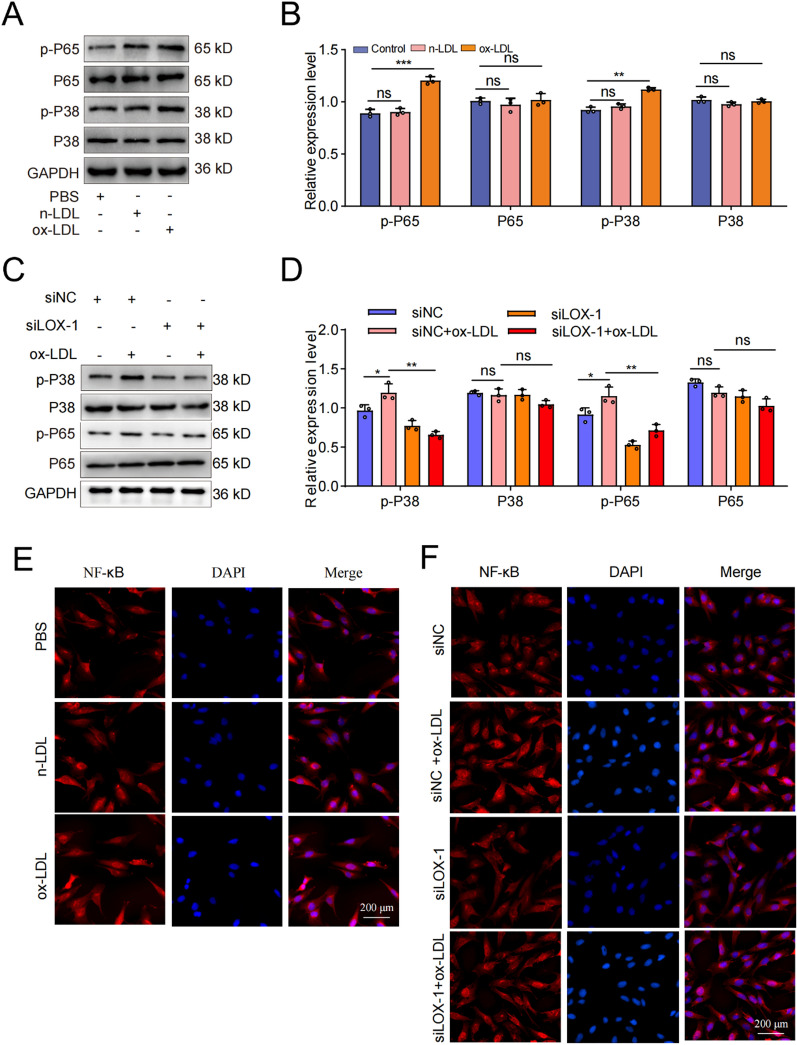
The P38-MAPK/NF-κB signaling pathway was involved in LOX-1/ox-LDL -induced cartilage endplate cells senescence. **A**, **B** Western blotting and quantitative analysis were performed to assess the expression levels of pathway-related proteins following ox-LDL treatment. **C**,** D** Western blotting and quantitative analysis were conducted to evaluate the expression levels of pathway-related proteins after treatment with ox-LDL and siLOX-1/siNC. **E** The results of immunofluorescence experiment also indicated that ox-LDL treatment could promote the nuclear translocation of NF-κB. **F** siLOX-1 effectively inhibited the promoting effect of ox-LDL on nuclear translocation of NF-κB. Compared to siNC/ control group, **p < 0.01, ***p < 0.001, ns indicated non-significance difference; Compared to siNC + ox-LDL group, *p < 0.05, **p < 0.01, ns indicated non-significance difference

Previous studies have demonstrated the pivotal role of MAPK/NF-κB signaling pathway in ameliorating aging and calcification in rats (Xu et al. 2021; Han et al. 2019). In this study, our objective was to further validate the involvement of the P38-MAPK/NF-κB signaling pathway in these processes. Firstly, we used WB experiment to investigate the impact of ox-LDL treatment on the expression of pathway-related proteins, as depicted in Fig. 8A, B. From these figures, it was evident that ox-LDL treatment significantly upregulated the expression of p-P65 and p-P38 proteins while exerting minimal influence on the total levels of P65 and P38 proteins. This indicated that ox-LDL could regulate the P38-MAPK/NF-κB signaling pathway in EPCs. The results of immunofluorescence experiment also indicated that ox-LDL treatment could promote the nuclear translocation of NF-κB (Fig. 8E**)**. Subsequently, we examined alterations in protein expression associated with this pathway using ox-LDL and siLOX-1/siNC treatments and discovered that siLOX-1 treatment effectively inhibited the promoting effect of ox-LDL on the expression level of p-P65 and p-P38 proteins (Fig. 8C, D). This inhibition was further confirmed by observing the nuclear translocation of NF-κB in immunofluorescence assay (Fig. 8F**)**. Consequently, we concluded that the activation of the P38-MAPK/NF-κB signaling pathway was implicated in the senescence induced by ox-LDL/LOX-1 in EPCs.

### The ROS/P38-MAPK/NF-κB signaling pathway was implicated in the regulation of EPCs senescence induced by ox-LDL/LOX-1

**Fig. 9:**
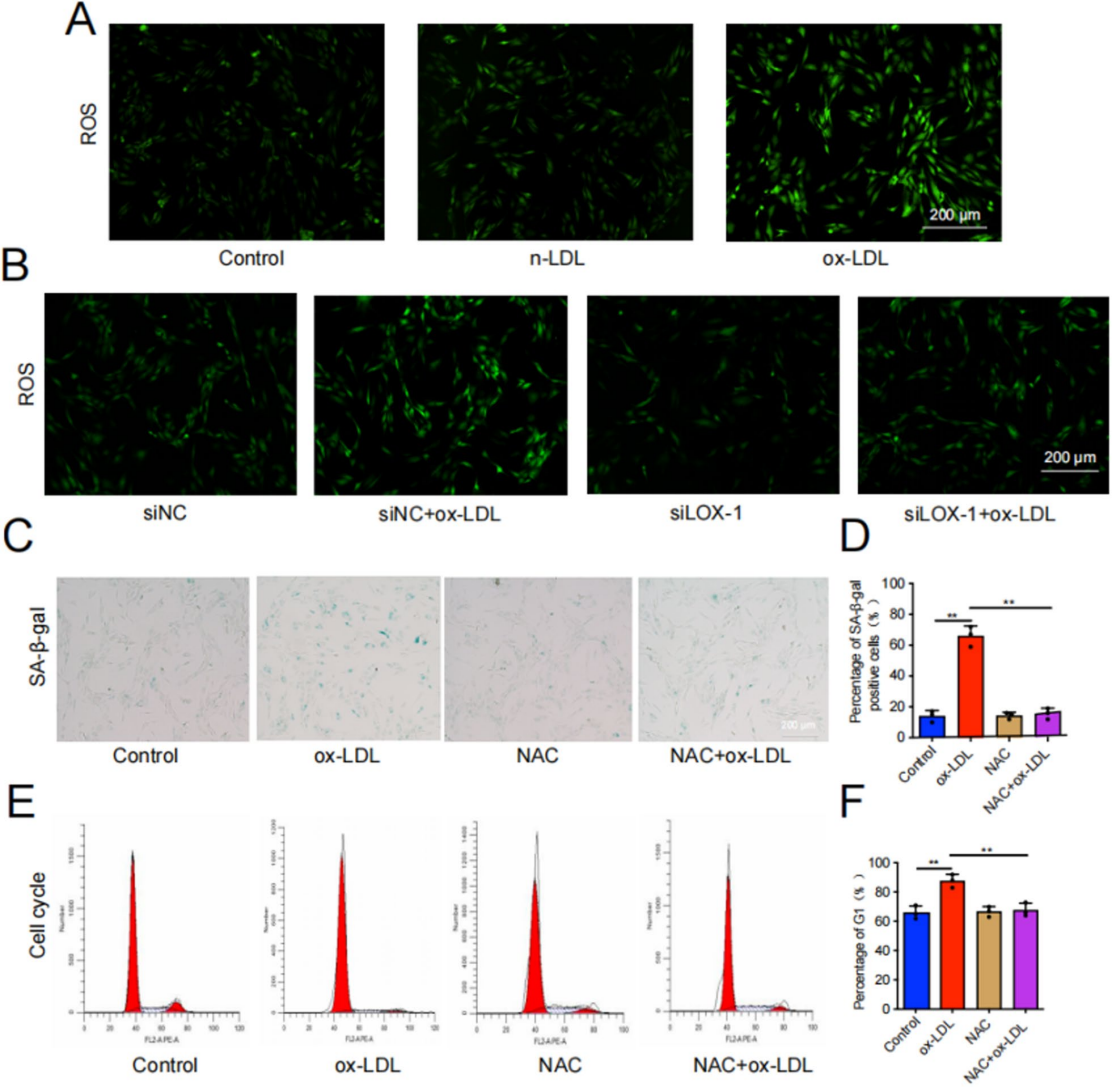
ROS was implicated in the regulation of cartilage endplate cells senescence induced by ox-LDL/LOX-1. **A**,** B** DCFH-DA fluorescence staining was performed to detect ROS levels (n = 3, scale = 200 μm). **C**,** D** SA-β-Gal staining results and quantitative analysis were conducted to assess cellular senescence (Scale bar = 200 μm). **E**,** F** Cell cycle detection and analysis results. Compared to ox-LDL group, *p < 0.05, **p < 0.01, ns indicated non-significance difference

**Fig. 10:**
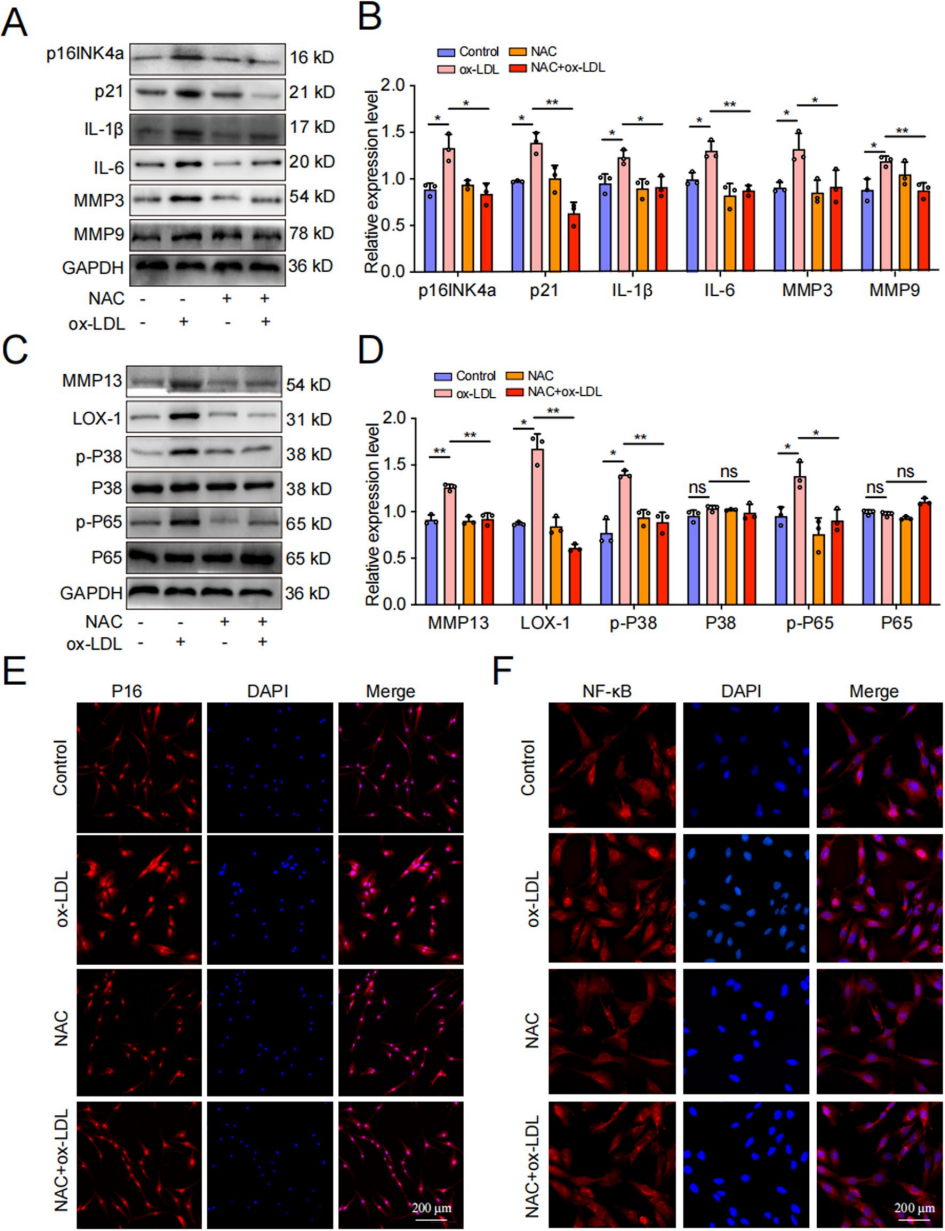
ROS/P38-MAPK/NF-κB signaling pathway was implicated in the regulation of cartilage endplate cells senescence induced by ox-LDL/LOX-1. **A**-**D** Western blot and quantitative analysis were performed to evaluate the expression of cell senescence/calcification-related proteins as well as P38-MAPK/NF-κB pathway-related proteins. **E**, **F** P16 and NF-κB representative immunofluorescence staining results. Compared to ox-LDL group, *p < 0.05, **p < 0.01, ns indicated non-significance difference

**Fig. 11:**
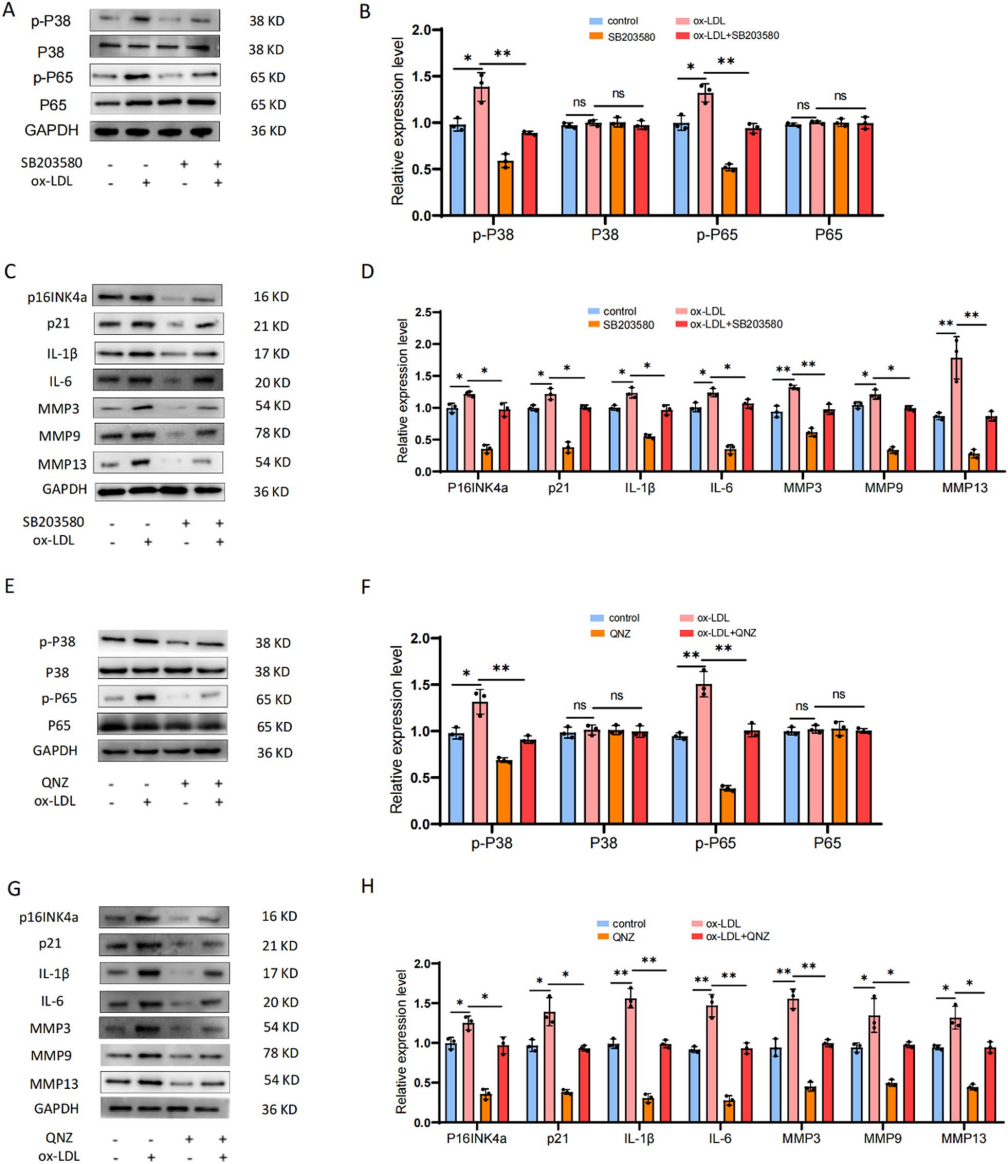
P38 inhibitor (SB203580) and NF-κB inhibitor (QNZ) were use to further identify that P38-MAPK/NF-κB were implicated in the regulation of cartilage endplate cells senescence induced by ox-LDL/LOX-1. **A**-**D** SB203580 was used to further identify that P38-MAPK were implicated in the regulation of cartilage endplate cells senescence induced by ox-LDL/LOX-1. **E**–**H** QNZ was use to further identify that NF-κB was implicated in the regulation of cartilage endplate cells senescence induced by ox-LDL/LOX-1. Western blot and quantitative analysis were performed to evaluate the expression of cell senescence related proteins as well as P38-MAPK/NF-κB pathway-related proteins. Compared to control group, *p < 0.05, **p < 0.01, ns indicated non-significance difference. Compared to ox-LDL group, *p < 0.05, **p < 0.01, ns indicated non-significance difference

Oxidative stress is now recognized as a trigger factor in cellular aging, with ROS being the primary instigator (Hajam et al. 2022; Liguori et al. 2018). Firstly, we assessed the ROS level in each treatment group through DCFH-DA fluorescence staining, and observed a significant increase of ROS levels in EPCs following ox-LDL treatment. However, siLOX-1 was found to attenuate the impact of ox-LDL treatment on ROS levels in EPCs (Fig. 9A, B). In order to further verify whether the ROS/P38-MAPK/NF-κB pathway was involved in LOX-1/ox-LDL-induced EPCs senescence and calcification, we pre-treated EPCs with a ROS inhibitor (NAC), P38 inhibitor (SB203580), and an NF-κB inhibitor (QNZ) before exposing them to ox-LDL. β-galactosidase staining, flow cytometry, WB, and cell immunofluorescence were used to evaluate senescence, calcification, and P38-MAPK/NF-κB pathway-related indicators. Our results demonstrated that compared to the ox-LDL group, the ox-LDL + NAC group exhibited significantly reduced SA-β-Gal-positive staining cells and G1 phase cells in the cell cycle (Fig. 9C–F). WB analysis revealed that NAC effectively inhibited the upregulation of cell senescence related proteins induced by ox-LDL (Fig. 10A–D). The results of cell immunofluorescence further confirmed the reliability of the WB results, showing highly consistency (Fig. 10E, F). Additionally, SB203580 and QNZ effectively inhibited the promoting effect of ox-LDL on cell senescence-related proteins **(**Fig. 11A–H**)**. To sum up, our data confirmed that LOX-1 activation induced by ox-LDL led to senescence of EPCs via the ROS/P38-MAPK/NF-κB signaling pathway.

## Discussion

Recent studies have demonstrated that lipid metabolism disorders, such as HLP, can significantly increase the risk of IDD and exacerbate its progression, establishing HLP as an independent risk factor for IDD (Ruiz-Fernández et al. 2019; Francisco et al. 2022). However, the underlying molecular mechanisms of this process remain incompletely understood. In this study, we provided a comprehensive analysis of the impact of lipid metabolism disorders on IDD, with a particular focus on EPCs senescence and calcification. Our findings revealed a significant upregulation in the expression of ox-LDL/LOX-1, accompanied by increased senescence and calcification phenotypes in EPCs during the progression of IVD. In addition, our investigation uncovered a positive correlation between ox-LDL/LOX-1 expression and the severity of HLP-induced CEP degeneration. We highlighted the role of LOX-1 in mediating ox-LDL-induced senescence and calcification in EPCs. Intriguingly, we identified that the induced effects of ox-LDL/LOX-1 on EPCs’ senescence and calcification could be mitigated by blocking the ROS/P38-MAPK/NF-κB signaling pathway.

Studies have shown that dysregulation of pro-inflammatory adipokines related to lipid metabolism and elevated levels of circulating lipids contribute to cellular senescence, apoptosis and extracellular matrix (ECM) degradation in IVD, thereby accelerating the progression of IDD. For instance, Zhang et al. (Zhang et al. 2019a) proved that HLP aggravated IDD by inducing inflammatory responses and catabolism of NP and AF cells. Another study found that HLP could induce IDD by promoting apoptosis in NP cells and enhancing ECM catabolism (Zhang et al. 2019b). Although it has been confirmed that lipid metabolism disorders can induce IDD, the animal model used to study these disorders have not been stable or accurate enough, and their reliability has not been fully verified. In this study, we successfully established a reliable rat model of IDD induced by lipid metabolism disorders, and comprehensively validated its accuracy by MRI, micro-CT scanning, proteomics analysis and tissue immunofluorescence. Our findings strongly support a close association between lipid metabolism disorders and IVD cell senescence, inflammatory response activation, as well as calcification processes. This provided a solid theoretical foundation for subsequent investigations into molecular mechanisms underlying these associations.

Several studies have demonstrated that ox-LDL mediates LOX-1, inducing senescence and calcification of smooth muscle cells and endothelial cells, thereby promoting the development of atherosclerosis (Song et al. 2017; Yamashita et al. 2020; Li et al. 2022b). Additionally, ox-LDL/LOX-1 has been implicated in the pathogenesis of osteoarthritis, potentially contributing to oxidative stress injury, senescence, and calcification of chondrocytes (Akagi et al. 2009; Kishimoto et al. 2010; Nishimura et al. 2004). Recent research has revealed high levels of ox-LDL expression in degenerative IVD tissues, suggesting a potential involvement of ox-LDL/LOX-1 in the pathogenesis of IDD. However, further exploration is required to fully understand the underlying mechanisms (Li et al. 2017; Wu et al. 2021). Our research focused on investigating the impact of ox-LDL/LOX-1 on CEP, which are early perceiver and victims of lipid metabolism disorders. Our study exhibited elevated expression levels of ox-LDL/ LOX-1 in degenerative CEP tissues in both clinical samples and animal models, implying a correlation between the expression level of ox-LDL/LOX-1 and the degree of CEP degeneration.

Studies have shown that CEP degeneration may induce IDD through the secretion of inflammatory cytokines by senescent cells, upregulation of gene expression and calcification of matrix degrading enzymes (Kong et al. 2023; Xie et al. 2020). Previous clinical studies have demonstrated a positive correlation between serum lipids levels and the extent of CEP degeneration. However, there is a lack of fundamental research on the effect of lipid metabolism disorder on CEP degeneration. Therefore, our study aimed to investigate the effect and molecular mechanism of lipid metabolism disorder on CEP degeneration. Further detections on HLP rats revealed that lipid metabolism disorders markedly promoted the expression of ox-LDL/LOX-1, while also inducing calcification and senescence of EPCs. Proteomic analyses further identified enrichment in cell cycle control and endochondral bone morphogenesis signaling pathways. These findings prompted us to explore whether elevated ox-LDL/LOX-1 expression contributes to EPCs senescence and CEP calcification. Consequently, we conducted follow-up research to delve deeper into this hypothesis.

A substantial body of evidence has demonstrated that ox-LDL/LOX-1 can induce senescence and the SASP in smooth muscle cells and endothelial cells (Barreto et al. 2021; He et al. 2021; Zhang et al. 2021). Jia et al. (Silwal et al. 2023) have shown that LOX-1 mediates lipid accumulation and senescence in macrophages induced by ox-LDL, resulting in increased expression of p53, p21 and p16 proteins. Hashimoto et al. (Yin et al. 2021) have found that ox-LDL/LOX-1 participates in the development of arthritis by promoting articular cartilage calcification and cartilage matrix degradation. Our study discovered that high concentrations of ox-LDL could inhibit the viability of EPCs, significantly enhance the expression of senescence and calcification genes, and block the cell cycle at G1 phase. It was worth noting that ox-LDL also promoted the formation of calcium nodules. Subsequently, we further explored the role of the LOX-1 receptor in this process. The detection methods including β-Galactosidase staining, flow cytometry, WB and cellular immunofluorescence collectively confirmed that ox-LDL-induced senescence and calcification of EPCs were dependent on the LOX-1 receptor, which was consistent with the reports from other researchers.

The P38-MAPK/NF-κB signaling pathway, a classic pathway, has been confirmed to regulate cellular senescence, and plays a crucial role in creating a microenvironment conducive to this process (Jia et al. 2019; Hashimoto et al. 2016). Under the condition of oxidative stress, excessive ROS can damage cellular proteins and DNA, leading to cell impairment, senescence, as well as other related phenomena. Nishimura et al. (Nishimura et al. 2004) have reported that ox-LDL/LOX-1 promotes intracellular ROS production, activates the NF-κB signaling pathway, and induces catabolism and inflammatory response of chondrocytes. Our observations fully support this conclusion. We found that the ROS level in EPCs was significantly increased after ox-LDL treatment. However, transfection with siLOX-1 could mitigate the effect of ox-LDL on ROS levels in EPCs as demonstrated by DCFH-DA fluorescent staining. Furthermore, our experimental results using the ROS inhibitor and pathway inhibitors, along with the detection of pathway-related proteins, provided additional evidence for the involvement of the ROS/P38-MAPK/NF-κB signaling pathway in regulating ox-LDL/LOX-1 induced senescence and calcification in EPCs.

Despite the aforementioned findings, this investigation still possesses several limitations. We have yet to determine the causes of elevated oxidative stress and whether it is associated with mitochondria disfunction. Furthermore, the impact of ox-LDL/LOX-1 on mitochondria and other organelles in EPCs has not been elucidated. Therefore, future research should prioritize these areas for a more comprehensive understanding.

## Conclusions

**Fig. 12:**
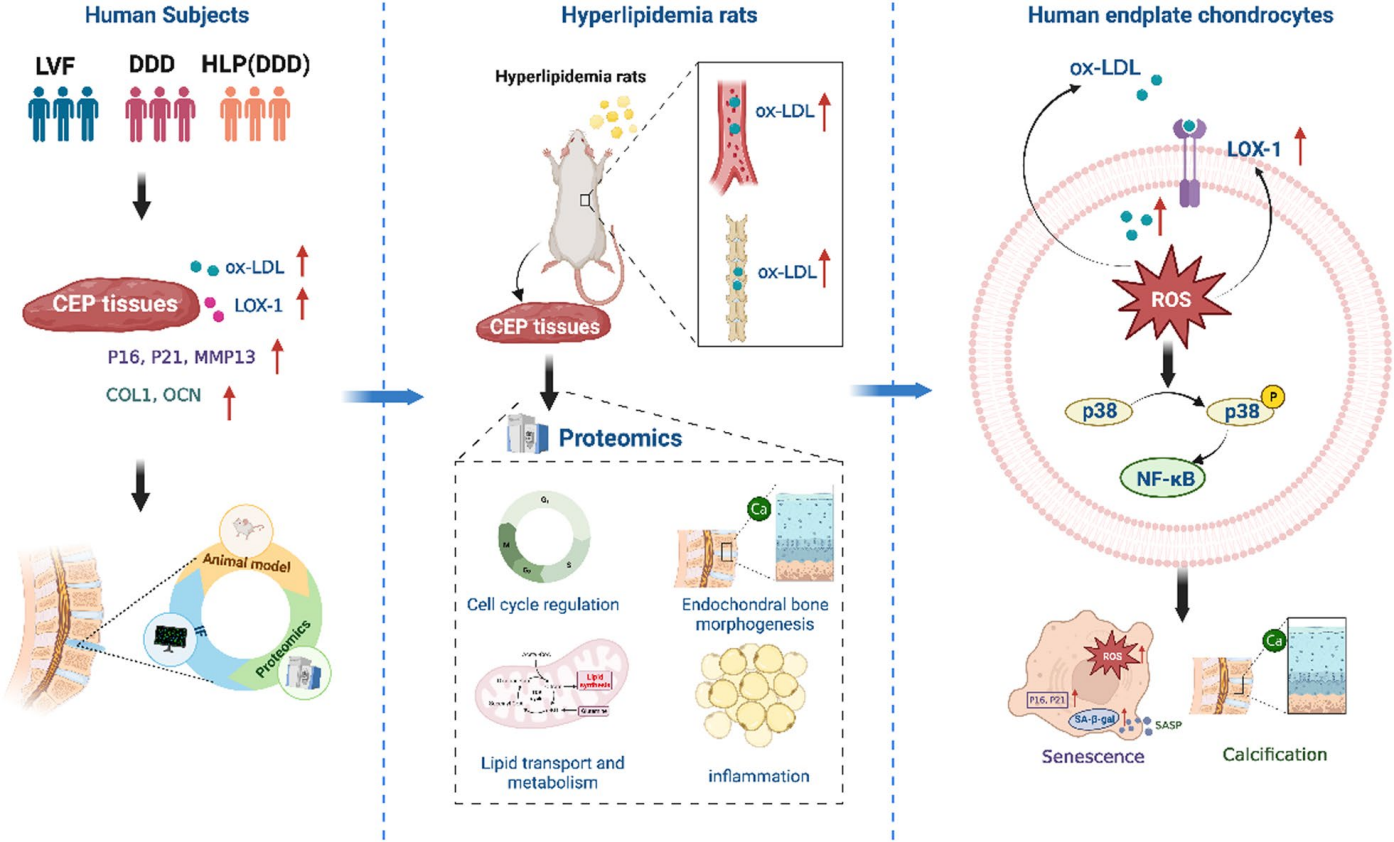
ox-LDL/LOX-1 induced senescence and calcification of cartilage endplate cells through ROS/P38-MAPK/NF-κB signaling pathway

In conclusion, our study has revealed a novel molecular mechanism underlying lipid metabolism disorder-induced IDD. For the first time, we demonstrated that lipid metabolism disorders could trigger senescence and calcification of EPCs via ox-LDL/LOX-1, ultimately leading to IDD. In addition, we elucidated that ox-LDL/LOX-1 induced senescence and calcification of EPCs through ROS/P38-MAPK/NF-κB signaling pathway (Fig. 12). In summary, our study provided new insights into the relationship between lipid metabolism disorders and IDD, uncovering a novel mechanism that may offer a potential therapeutic target for delaying cellular senescence in IVD.

### Supplementary Information

Supplementary Material 1.

Supplementary Material 2.

## Data Availability

The authors confirm that the data supporting the findings of this study are available within the article and its supplementary materials.

## Abbreviations

HLP: Hyperlipidaemia
IDD: Intervertebral Disc Degeneration
ox-LDL: Oxidized low density lipoprotein
LOX-1: Lectin-like oxidized low-density lipoprotein receptor 1
IVD: Intervertebral disc
NP: Nucleus pulposus
AF: Annulus fibrosus
CEP: Cartilage end plates
MRI: Magnetic resonance imaging
PBS: Phosphate buffered saline
DMEM: Dulbecco's modified Eagle's medium
LVF: Lumbar vertebral fractures
OCN: Osteocalcin
ROS: Reactive oxygen species
ELISA: Enzyme-linked immunosorbent assay
Runx2: Runt-related transcription factor 2
H&E: Hematoxylin–eosin
SD: Sprague–Dawley
SASP: Senescence associated secretory phenotype
LDL-C: Low density lipoprotein-cholesterol
NAC: N-acetylcysteine
WB: Western blot
ALP: Alkaline phosphatase staining
ECM: Extracellular matrix
HFD: High-fat diet
