# Supplementary figures and images for: Dysregulated lipid metabolism and intervertebral disc degeneration: the important role of ox-LDL/LOX-1 in endplate chondrocyte senescence and calcification
Source: Mol Med. 2024 Aug 9;30:117. doi: 10.1186/s10020-024-00887-8 (PMC11311918; doi:10.1186/s10020-024-00887-8)

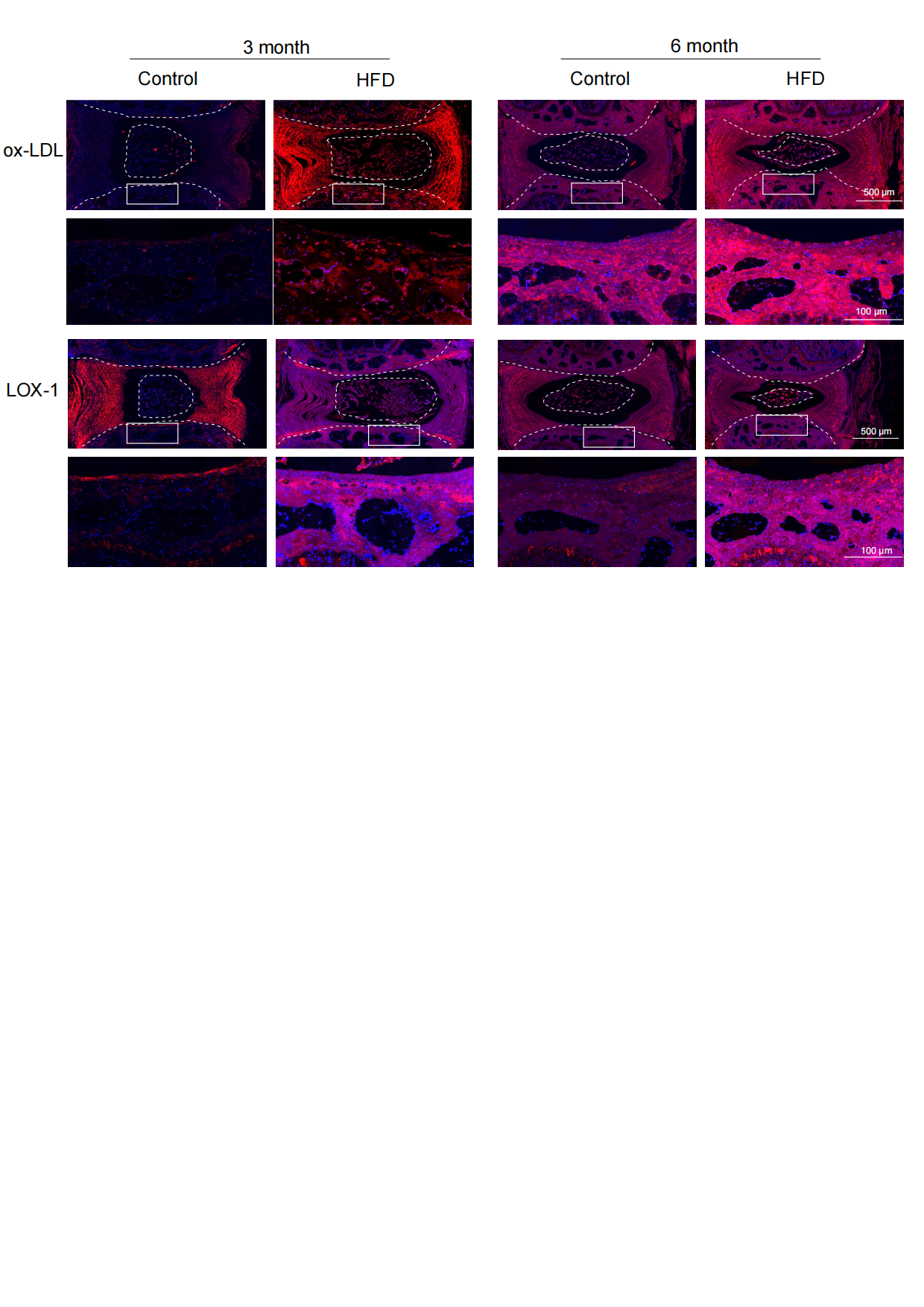

Supplement: Supplementary file 1 — Supplementary Material 1. [file 10020_2024_887_MOESM1_ESM.tif]

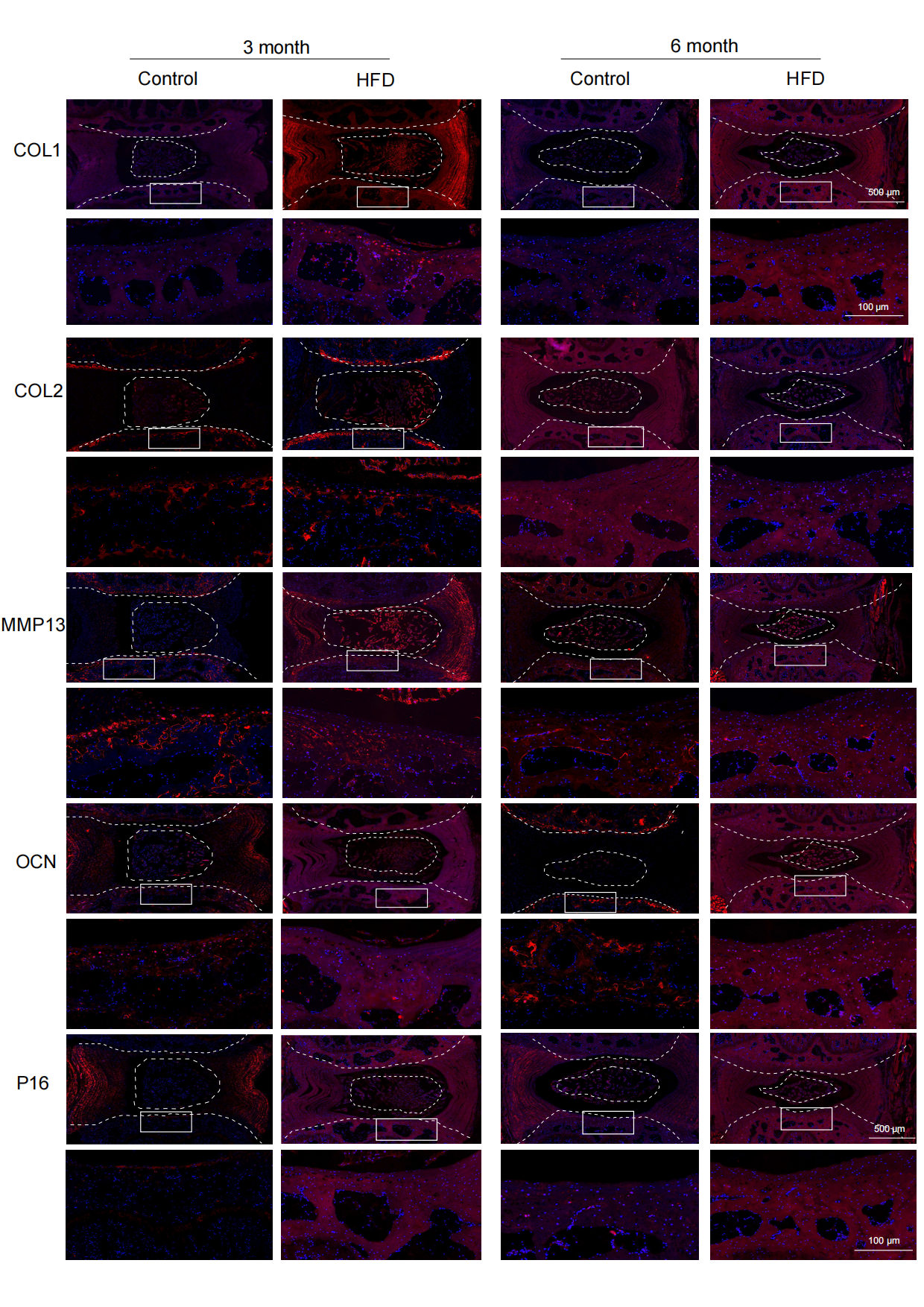

Supplement: Supplementary file 2 — Supplementary Material 2. [file 10020_2024_887_MOESM2_ESM.tif]
